# Prediction of Prehypertenison and Hypertension Based on Anthropometry, Blood Parameters, and Spirometry

**DOI:** 10.3390/ijerph15112571

**Published:** 2018-11-16

**Authors:** Byeong Mun Heo, Keun Ho Ryu

**Affiliations:** 1 Database/Bioinformatics Laboratory, Chungbuk National University, Cheongju 28644, Korea; muni4344@gmail.com; 2 Faculty of Information Technology, Ton Duc Thang University, Hochiminh City 700000, Vietnam; 3 Department of Computer Science, Chungbuk National University, Cheongju 28644, Korea

**Keywords:** machine learning, feature selection, hypertension, prehypertension, anthropometry, spirometry

## Abstract

Hypertension and prehypertension are risk factors for cardiovascular diseases. However, the associations of both prehypertension and hypertension with anthropometry, blood parameters, and spirometry have not been investigated. The purpose of this study was to identify the risk factors for prehypertension and hypertension in middle-aged Korean adults and to study prediction models of prehypertension and hypertension combined with anthropometry, blood parameters, and spirometry. Binary logistic regression analysis was performed to assess the statistical significance of prehypertension and hypertension, and prediction models were developed using logistic regression, naïve Bayes, and decision trees. Among all risk factors for prehypertension, body mass index (BMI) was identified as the best indicator in both men [odds ratio (OR) = 1.429, 95% confidence interval (CI) = 1.304–1.462)] and women (OR = 1.428, 95% CI = 1.204–1.453). In contrast, among all risk factors for hypertension, BMI (OR = 1.993, 95% CI = 1.818–2.186) was found to be the best indicator in men, whereas the waist-to-height ratio (OR = 2.071, 95% CI = 1.884–2.276) was the best indicator in women. In the prehypertension prediction model, men exhibited an area under the receiver operating characteristic curve (AUC) of 0.635, and women exhibited a predictive power with an AUC of 0.777. In the hypertension prediction model, men exhibited an AUC of 0.700, and women exhibited an AUC of 0.845. This study proposes various risk factors for prehypertension and hypertension, and our findings can be used as a large-scale screening tool for controlling and managing hypertension.

## 1. Introduction

Hypertension is a major disease burden worldwide and an important risk factor for cardiovascular disease (CVD), chronic kidney disease (CKD) and death [1,2,3,4,5,6,7], and prehypertension is also a risk factor that can lead to CVD, stroke and CKD [8,9,10,11]. Hypertension is highly associated with poor diet, low physical activity and excessive alcohol consumption [7,12]. The treatment of hypertension reduces the risk of stroke, coronary artery disease, and congestive heart failure [1].

Methods for managing hypertension include antihypertensive therapy and exercise therapy. Antihypertensive therapy reduces the mortality rates associated with stroke, myocardial infarction, CVD and heart failure [13,14,15,16,17], and exercise therapy is effective for lowering systolic blood pressure (SBP), diastolic blood pressure (DBP), and the levels of total cholesterol (TC), low-density lipoprotein cholesterol (LDL-C), triglyceride (TG) and high-density lipoprotein cholesterol (HDL-C) [18,19,20,21,22].

The obesity indices have been mainly used in association studies with hypertension. For example, waist circumference (WC) is a risk factor for hypertension in African populations, Caribbean populations, Brazilian women, and Filipina women [23,24,25,26]. The body mass index (BMI) is a risk factor for hypertension in China, the Philippines, the United States (US), Australian women and India [27,28,29]. The WC ratio (WHR) is associated with hypertension in Chinese women in Hong Kong and in Australian men [29,30], and the waist-to-height ratio (WHTR) is the best indicator of hypertension in Chinese men in Hong Kong [30], a Taiwanese population [31] and a Korean population [32]. WC, BMI, and WHR are also associated with prehypertension in a Taiwanese population [33].

Several published studies have investigated blood parameters and hypertension. For example, compared with normotensive individuals, hypertensive patients show higher levels of fasting plasma glucose, serum high-sensitivity C-reactive protein (hs-CRP), TG, TC, LDL-C, uric acid (UA), white blood cells (WBCs), red blood cells (RBCs), hemoglobin (HGB), hematocrit (HCT) and mean corpuscular hemoglobin and lower serum HDL-C, mean corpuscular volume and RBC distribution width [34,35]. A higher glycated hemoglobin (HbA1c) level is correlated with a higher prevalence of hypertension [36], and inadequate hypertension treatment elevates serum creatinine level [37]. Markers of inflammation (CRP, WBC, amyloid-a, and homocysteine) are present at high levels in men and women with prehypertension [38].

In previous studies of spirometry and hypertension, the forced vital capacity (FVC) was identified as a negative predictor of hypertension, and lower FVC values were found to be a risk factor for future hypertension [39,40]. In studies of Beijing and Guangzhou populations, the FVC and forced expiratory volume in 1 s (FEV1) were found to be inversely proportional to SBP and DBP in women in both populations and in men in Beijing. A follow-up study conducted 2 or 4 years later showed a low incidence of hypertension with low lung function, but this effect was found only in Guangzhou women [41]. In Swedish men aged 55–68 years, BP increased with decreasing FVC. A lower FEV1 was correlated with higher SBP and DBP [42].

Previous studies have reported the associations of anthropometry, blood parameters, and spirometry with hypertension or prehypertension, but no studies have yet described the relationships between each of prehypertension and hypertension and anthropometric indices, blood parameters, and spirometric indices. The purposes of this study are to analyze risk factors of hypertension and prehypertension and to present a machine-learning-based prediction model to reduce the risks of diseases (CVD, CKD, stroke) caused by hypertension and to prevent diseases. First, we present risk factors for hypertension and prehypertension with statistical significance using demographic indices, anthropometric indices, blood parameters, and spirometric indices. Second, we develop predictive models of hypertension and prehypertension based on machine learning using correlation-based feature selection (CFS) and wrapper-based feature selection (WFS) methods and logistic regression (LR), naïve Bayes (NB), and decision tree (DT) prediction algorithms. Last, we propose the best hypertension and prehypertension prediction model through the performance evaluation between the developed prediction models. To the best of our knowledge, this study provides the first demonstration of the associations of prehypertension and hypertension with obesity indices, blood parameters, and spirometric indices in a Korean population. The findings of the present study provide basic information for the treatment and prevention of prehypertension and hypertension.

## 2. Materials and Methods

### 2.1. Subjects and Dataset

We obtained data from the sixth Korea National Health and Nutrition Examination Survey (KNHANES VI). The KNHANES database is publicly available at the KNHANES website (http://knhanes.cdc.go.kr/knhanes/eng). In this study, demographics, anthropometric indices, blood parameters and spirometric indices from the KNHANES VI were analyzed. KNHANES was approved by the Institutional Review Board of the Korea Centers for Disease Control & Prevention (KCDC) and the KCDC Bioethics Committee. Informed consent was obtained from all the participants prior to KNHANES data collection (Approval Numbers: 2013-07CON-03-4C, 2013-12EXP-03-5C and 2015-01-02-6C).

The total number of subjects was 22948. According to the exclusion criteria, we performed the following:

- We selected 12,838 individuals after excluding 10110 participants under 40 years of age.
- We excluded 4626 patients with missing values (FVCP, *n* = 4008; HDL-C, *n* = 585; HbA1c, *n* = 30; WC, *n* = 1; and hypertension diagnosis, *n* = 2).

**Figure 1:**
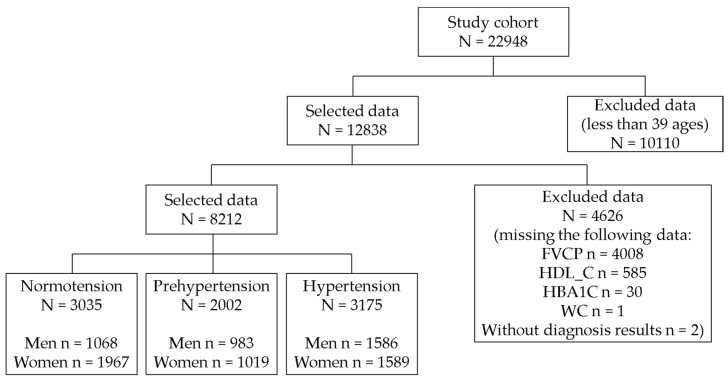
Sample selection procedure.

In total, 8212 subjects were included in the study. According to the definitions of hypertension, 3035 subjects with normal blood pressure, 2002 subjects with prehypertension and 3175 subjects with hypertension were classified (the details of the data preprocessing are provided in Figure 1).

### 2.2. Definitions of Prehypertension and Hypertension

We used the criteria proposed by the World Health Organization (WHO) [5], the Joint National Committee 7 (JNC 7) [43] and previous studies to define normotension, prehypertension and hypertension [8,10,11,33,44,45]. Normotension was defined as an SBP less than 120 mmHg and a DBP less than 80 mmHg. Prehypertension was defined as an SBP between 120 mmHg and 139 mmHg and a DBP between 80 mmHg and 89 mmHg. Hypertension was defined as an SBP of at least 140 mmHg and/or a DBP of at least 90 mmHg [43],a diagnosis of hypertension or reported use of antihypertensive medications [11].

### 2.3. Statistical Analysis

Statistical analyses were performed using SPSS 20 for Windows (SPSS Inc., Chicago, IL, USA). Binary logistic regression (LR) was conducted to identify significant differences between normotension and prehypertension and between normotension and hypertension after standardized transformation was applied to the male and female datasets. Independent two-sample t-tests were used to examine the differences between men and women (basic characteristics are described in Table 1). We developed prehypertension and hypertension prediction models using the Waikato Environment for Knowledge Analysis data mining tool. The prediction models were developed using LR, naïve Bayes (NB), and decision tree (DT) classification algorithms, all of which are widely used classification models. The NB classifier uses Bayes’ theorem and conditional probability to measure the probability of occurrence between classes and attributes and has the advantage of low computational cost [46]. The DT classifier creates an attribute with high information gain as an upper node based on entropy. Specifically, this classifier recursively creates an optimal tree structure by partitioning followed by pruning. DT has the advantages of being easy to understand, providing a visual tree structure and having low calculation cost [47]. LR classifiers are used extensively in medical statistical surveys because the results of analyses relating categorical dependent variables and one or more independent variables are easily interpreted. Depending on the number of dependent variables, a binary or polynomial model may be used [46]. To select the features associated with prehypertension and hypertension, correlation-based feature selection (CFS) and wrapper-based feature selection (WFS) were applied. CFS solves the multicollinearity problem to recommend a variable with low correlation between attributes and high correlation between attribute and class [48], and WFS selects variables through black-box testing using a classification algorithm [49].

### 2.4. Performance Evaluation

The prehypertension and hypertension predictive models were tested using the area under the receiver operating characteristic curve (AUC), and the performance of each predictive model was evaluated through analyses of sensitivity and (1-specificity). Sensitivity indicates that the response value was predicted to be positive in the positive case, and (1-specificity) is the false positive value in the negative case. The data were standardized such that the numerical data in different ranges could be analyzed on the same line. A ten-fold cross-validation test was performed to evaluate the predictive power of each model.

## 3. Results

The normal BP group included 1068 (13%) men and 1967 (24%) women, whereas the prehypertension group included 983 (12%) men and 1019 (12.4%) women, and the hypertension group included 1586 (19.3%) men and 1589 (19.3%) women. In the statistical analyses, *p*-values, odds ratios (ORs), and 95% confidence intervals (CIs) for each feature were obtained using binary LR. Table 2 and Table 3 show the significance of differences in the studied variables between normotension and prehypertension or hypertension after adjustment for age in men and women.

### 3.1. Statistical Analysis of Prehypertension

In men, BMI (OR = 1.429, 95% CI = 1.304–1.567) was identified as the best indicator of prehypertension. WT (OR = 1.365, 95% CI = 1.242–1.500) and WHTR (OR = 1.335, 95% CI = 1.219–1.462) were also significantly associated with prehypertension. Among the blood parameters, hemoglobin (HGB; OR = 1.323, 95% CI = 1.204–1.453) exhibited an association with prehypertension in men. No spirometric indices were significant. In women, BMI (OR = 1.428, 95% CI = 1.322–1.542) was found to be the best indicator, and WHTR (OR = 1.425, 95% CI = 1.313–1.546) and WC (OR = 1.386, 95% CI = 1.282–1.498) were also significantly associated with prehypertension. Among the blood parameters, glucose (GLU) (OR = 1.290, 95% CI = 1.180–1.410) was associated with prehypertension in women, and among the spirometric indices, the FVCP (OR = 0.814, 95% CI = 0.755–0.878) exhibited an association with prehypertension in women.

### 3.2. Statistical Analysis of Hypertension

In men, BMI (OR = 1.993, 95% CI = 1.818–2.186) was found to be the best indicator of hypertension, and WHTR (OR = 1.903, 95% CI = 1.735–2.087) and WC (OR = 1.790, 95% CI = 1.638–1.956) were also significantly associated with hypertension. Among the blood parameters, TG (OR = 1.434, 95% CI = 1.304–1.577) exhibited a significant association with hypertension in men, and among the spirometric indices, FVCP (OR = 0.791, 95% CI = 0.728–0.859) showed a significant association with hypertension in men. In women, WHTR (OR = 2.071, 95% CI = 1.884–2.276) was identified as the best indicator of hypertension, and BMI (OR = 2.034, 95% CI = 1.861–2.222) and WC (OR = 1.927, 95% CI = 1.764–2.105) were also significantly associated with hypertension. Among the blood parameters, GLU (OR = 1.676, 95% CI = 1.508–1.861) exhibited a significant association with hypertension in women. Among the spirometric indices, the FVCP (OR = 0.682, 95% CI = 0.629–0.739) was found to be significantly associated with hypertension in women.

### 3.3. Performance Evaluation of the Prehypertension Prediction Model Combined with Feature Selection

We developed prediction models for prehypertension and hypertension using feature selection methods and classification algorithms. Features were selected using the CFS and WFS methods, and the predictive models were developed by applying the LR, NB, and DT algorithms with the selected features. The AUC was used to evaluate the performance of each prediction model.

The analysis of the prehypertension prediction model revealed that the WFS-LR model with AGE, BMI, GLU, TC, HDL-C, TG, aspartate aminotransferase (AST), HGB and blood urea nitrogen (BUN) showed the best predictive power (AUC = 0.635) for men, with a sensitivity of 0.52 and a1-specificity of 0.338. In contrast, the CFS-DT model showed the lowest predictive power (AUC = 0.559). For women, the WFS-LR model with AGE, WHTR, BMI, GLU, TC, TG, WBC, RBC and FVCP and peak expiratory flow (PEF) showed the best predictive power (AUC = 0.700), with a sensitivity of 0.308 and a 1-specificity of 0.11, whereas the CFS-DT model exhibited the lowest predictive power (AUC = 0.622).

**Figure 2:**
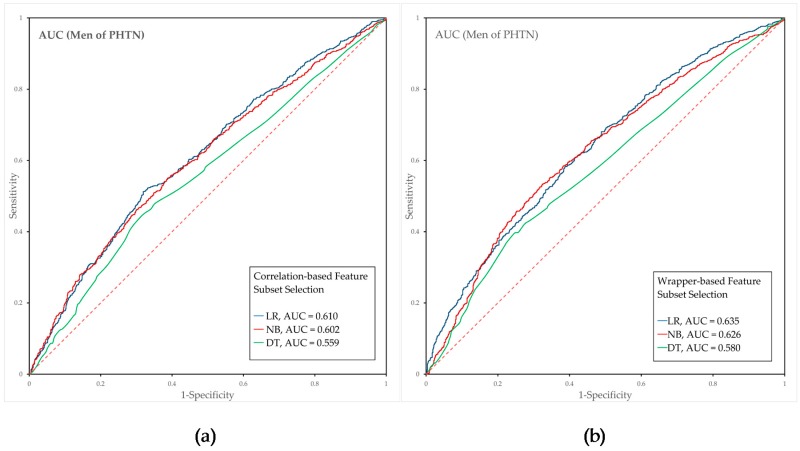

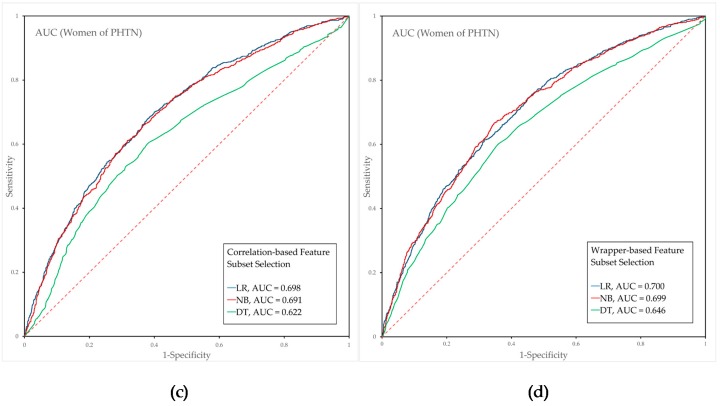
Predictive power of each prehypertension prediction model for men and women. Each graph presents the results for the LR, NB, and DT algorithms. (**a**,**b**) present the results for men, and (**c**,**d**) present the results for women. (**a**,**c**) show the results obtained with CFS, and (**b**,**d**) show the results obtained with WFS. Abbreviations: LR, logistic regression; NB, naïve Bayes; DT, decision tree; AUC, area under the receiver operating characteristic curve; PHTN, prehypertension; CFS, correlation-based Feature selection; WFS, wrapper-based feature selection.

The predictive performance of the prehypertension prediction model is compared and shown in Figure 2. The analyses of the prehypertension prediction model for men showed that the AUCs of LR, NB, and DT based on CFS were 0.610, 0.602 and 0.559, respectively, and that the AUCs of LR, NB, and DT based on WFS were 0.635, 0.626, and 0.580, respectively. In contrast, the AUCs of the prehypertension prediction model for women generated using LR, NB, and DT based on CFS were 0.698, 0.691 and 0.622, respectively, and the AUCs of LR, NB, and DT based on WFS were 0.700, 0.699 and 0.646, respectively.

### 3.4. Performance Evaluation of the Hypertension Prediction Models Combined with Feature Selection

Analyses of the hypertension prediction models revealed that the WFS-LR model with WHTR, BMI, GLU, TC, HDL-C, TG, AST, BUN, creatinine (CRT), WBC, FVC, the FEV1 to FVC ratio (FEV1FVC), FEV in 6 s (FEV6) and PEF showed the best predictive power (AUC = 0.777) for men. The sensitivity and 1-specificity of this model were 0.813 and 0.401, respectively. The CFS-DT model showed the lowest predictability (AUC = 0.666). For women, the WFS-LR model with AGE, WC, BMI, GLU, TC, HDL-C, TG, alanine aminotransferase (ALT), CRT, WBC, RBC, FVC, FEV6 and forced expiratory flow 25–75% (FEF 25–75) showed the best predictive power (AUC = 0.845), with a sensitivity of 0.724 and a 1-specificity of 0.191, and the CFS-DT model exhibited the lowest predictive power (AUC = 0.796).

**Figure 3:**
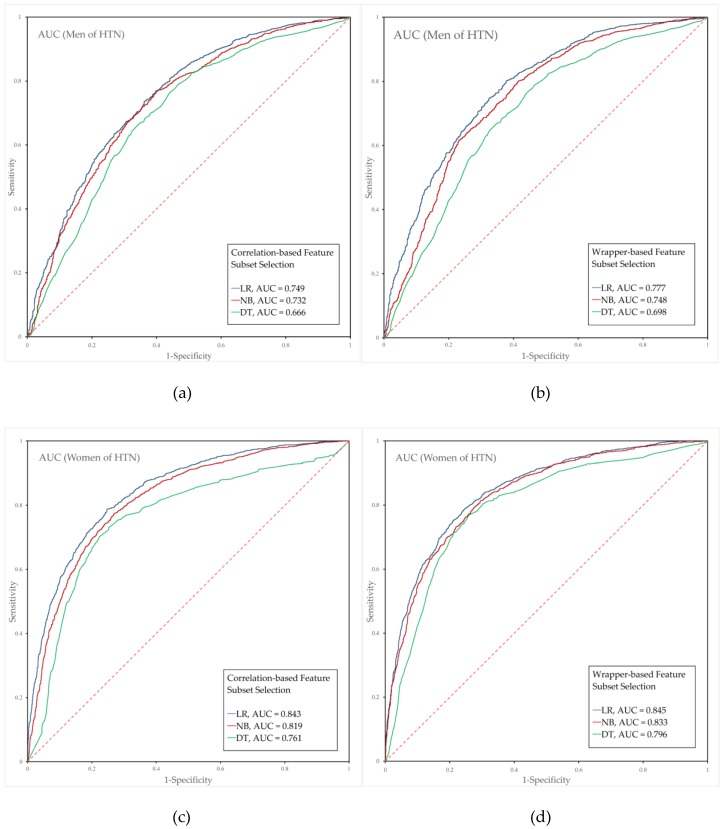
Predictive power of each hypertension prediction model for men and women. Each graph presents the results for the LR, NB, and DT algorithms. (**a**,**b**) present the results for men, and (**c**,**d**) present the results for women. (**a**,**c**) show the results obtained with CFS, and (**b**,**d**) show the results obtained with WFS. Abbreviations: LR, logistic regression; NB, naïve Bayes; DT, decision tree; AUC, area under the receiver operating characteristic curve; HTN, hypertension; CFS, correlation-based feature selection; WFS, wrapper-based feature selection.

The predictive performance of the hypertension prediction model is compared and shown in Figure 3. The analysis of the hypertension prediction model for men showed that the AUCs of LR, NB, and DT based on CFS were 0.749, 0.732 and 0.666, respectively, and that the AUCs of LR, NB, and DT based on WFS were 0.777, 0.748 and 0.698, respectively. In contrast, the AUCs of the hypertension prediction model for women generated through LR, NB, and DT based on CFS were 0.843, 0.819 and 0.761, respectively, and the AUCs of LR, NB, and DT based on WFS were 0.845, 0.833 and 0.796, respectively.

The features and performance results of the prehypertension and hypertension prediction models are summarized in Table 4. In men, the WFS-LR showed satisfactory performance (AUC = 0.635) in the prehypertension prediction model and the best performance (AUC = 0.777) in the hypertension prediction model. In contrast, in women, the WFS-LR showed satisfactory performance (AUC = 0.700) in the prehypertension prediction model and the best performance (AUC = 0.845) in the hypertension prediction model. Among the classification methods, LR exhibited higher prediction performance than did NB and DT. The hypertension prediction model performed better than the prehypertension prediction model and showed better performance in women than in men.

## 4. Discussion

In this study, anthropometric indices, blood parameters, and spirometric indices were examined to identify risk factors for prehypertension and hypertension. The features for the prehypertension and hypertension prediction models were selected using the CFS and WFS methods. Prediction models were then developed using the LR, NB, and DT classification algorithms.

In a previous study, Ko and colleagues analyzed the associations of BMI, WHR, WC, and WHTR with hypertension in a Chinese population in Hong Kong and found that WHTR was the strongest indicator in men (OR = 1.18, 95% CI = 1.14–1.23) whereas WHR was the strongest indicator in women (OR = 1.26, 95% CI = 1.18–1.35) [30]. Lee and colleagues demonstrated that among obesity factors, WC, WHR, and WHTR, were more predictive of hypertension than was BMI and that WHTR was the best obesity-related predictor of hypertension, regardless of gender, ethnicity and age [hazard ratio (HR) = 1.49, 95% CI = 1.35–1.65 in men and HR = 1.48, 95% CI = 1.33–1.64 in women] in middle-aged Korean adults [32]. Chang and colleagues found that BMI in men (OR = 2.07, 95% CI = 1.44–2.99) and abdominal obesity in women (OR = 2.04, 95% CI = 1.54–2.71) were associated with an increased risk of prehypertension [45]. Grievink and colleagues evaluated BMI, WC, and WHR as predictors of hypertension in a Caribbean population and identified WC (OR = 1.7, 95% CI = 1.4–2.0) as the best independent predictor of hypertension [24]. Tsai and colleagues reported that WHR, BMI, and WC were associated with prehypertension, particularly high BMI in men (OR = 1.106, 95% CI = 1.051) and high WC in women (OR = 1.031, 95% CI = 1.012–1.051) [33]. In this study, BMI was identified as the best predictor of prehypertension in men (OR = 1.429, 95% CI = 1.303–1.567) and women (OR = 1.427, 95% CI = 1.321–1.542). The risk factors that best predicted hypertension were BMI in men (OR = 1.993, 95% CI = 1.817–2.185) and WHTR in women (OR = 2.071, 95% CI = 1.884–2.276). Our findings are consistent with those of previous studies [27,28,29] and indicate that BMI is the best indicator of hypertension in men and of prehypertension in men and women.

Several studies of blood parameters and hypertension have been conducted. Cirillo and colleagues reported that hematocrit level was positively correlated with SBP and DBP in men and women [34]. In addition, Emamian and colleagues performed a multivariate LR analysis of demographic, biochemical, and hematological parameters and found that hematocrit (OR = 1.02, 95% CI = 1.003–1.04) was an independent predictor of hypertension [35]. Daniel and colleagues demonstrated that high HbA1c levels were associated with increased hypertension rate and that the rate of CVD (OR = 1.39, 95% CI = 1.06–1.83) increased by 1% with each increase in HbA1c level [36]. Christina and colleagues showed that men and women with prehypertension presented 31% higher CRP, 32% higher tumor necrosis factor-a, 9% higher amyloid-a, 6% higher homocysteine, and 10% higher WBC levels [38]. In this study, the best predictor of prehypertension was found to be HBG (OR = 1.322, 95% CI = 1.204–1.452) in men and GLU (OR = 1.289, 95% CI = 1.180–1.410) in women. HCT (OR = 1.262, 95% CI = 1.151–1.383) and TG (OR = 1.259, 95% CI = 1.162–1.365) were also highly associated with prehypertension in men and women, respectively. The best predictor of hypertension was TG (OR = 1.434, 95% CI = 1.304–1.576) in men and GLU (OR = 1.675, 95% CI = 1.508–1.861) in women. GLU (OR = 1.363, 95% CI = 1.247–1.489) and HbA1c (OR = 1.539, 95% CI = 1.393–1.700) were also highly associated with hypertension in men and women, respectively. Our findings are consistent with those of previous studies [36] and indicate that the HbA1c index is significantly associated with hypertension in women.

Through a study of hypertension and spirometry, Sarah and colleagues demonstrated that FVC was significantly associated with hypertension and a negative predictor [39]. Follow-up studies showed that hypertension could develop in the future, and an OR of approximately 0.7 was found in an LR analysis [39]. Jacobs and colleagues performed an HR analysis and found that the risk of hypertension (HR from 1 to 2.21) increased by more than 2-fold with decreasing FVC and that a low FVC might result in cardiovascular morbidity and mortality [40]. In this study, FVCP was identified as the best predictor of prehypertension and hypertension. Low FVCP indices were associated with prehypertension in women (OR = 0.814, 95% CI = 0.755–0.877), hypertension in men (OR = 0.791, 95% CI = 0.728–0.859) and hypertension in women (OR = 0.681, 95% CI = 0.629–0.739). FVC predictors were also significantly associated with hypertension, but the associations were slightly less significant than the association of FVCP with hypertension. Our findings are consistent with those of previous studies [39,40] and indicate that the FVC index is significantly associated with prehypertension in women and hypertension in men and women.

Prior to the present study, several researchers have proposed hypertension prediction model based on data mining techniques [50,51,52,53]. For instance, Tayefi and colleagues proposed a hypertension prediction model based on DTs in the Iranian population. The hypertension DT model suggested that demographics and selected biochemical markers (such as age, BMI, fasting blood GLU, TG, UA, hs-CRP, TC and LDL-C) have higher predictive power than other biochemical markers [50]. Ture and colleagues compared the performance of DTs, statistical algorithms, and neural networks using features such as age, sex, family history, smoking habits, lipoprotein, TG and UA and found that the neural network algorithm had the best predictive power for hypertension [51]. The evaluation of the performance of DT, NB, and LR performed in this study identified LR as the best classification algorithm. The model combining the demographic index, blood parameters and spirometric indices showed the best predictive power. Among the prediction models of prehypertension and hypertension, the WFS-LR prediction models were identified as the best for both men and women. A hypertension prediction model was then developed by combining the obesity index, blood parameters, and spirometric indices, whereas previous studies [50,51] used demographic characteristics, BMI, and blood parameters.

This study has several limitations. First, it is difficult to identify cause-and-effect relationships because we used data from a cross-sectional survey. Second, the most significant risk factor for hypertension was the obesity index, but hip circumference was not measured and could not be compared to WHR. Third, we did not have information on disease (diabetes, dyslipidemia, and hyperlipidemia) secondary to hypertension, so we did not consider it in this study. Finally, in this study, the predictive model was designed considering only anthropometric indices, blood parameters, and spirometric indices, and indicators such as smoking, drinking, and physical activity were excluded.

## 5. Conclusions

Hypertension is a risk factor that can lead to cardiovascular diseases and death, and treatment and management strategies for hypertension remain lacking. In this study, we examined the associations of prehypertension and hypertension with spirometric indices, obesity indices, and blood parameters and proposed prehypertension and hypertension prediction models to aid the effective management and prevention of hypertension. A statistical analysis of the three types of variables revealed that the obesity indices were the highest risk factors for prehypertension and hypertension in men and women. GLU, HbA1c, and TG as well as low spirometric values were also associated with hypertension in both men and women. Thus, we developed prediction models with the LR, NB and DT classifiers using two subset selection methods, namely, CFS and WFS. The predictive model with the highest prediction power was the WFS-LR prediction model that combined various factors (i.e., age, obesity indices, blood parameters, and spirometric indices). Our findings can be applied as a large-scale screening tool for the control and management of hypertension.

## Figures and Tables

**Table 1:** Basic characteristics of the subjects included in this study.

Features	Men (Mean SD)	Women (Mean SD)	Description
SUBJECTS	3637	4575	Number of subjects
AGE **	57.38 (10.78)	56.68 (10.34)	Years of age
HT ***	168.57 (6.21)	155.78 (5.8)	Height
WT ***	69.36 (10.17)	58.19 (8.3)	Weight
WC ***	85.88 (8.31)	80.17 (8.79)	Waist circumference
WHTR ***	0.5 (0.05)	0.51 (0.06)	Waist-to-height circumference ratio
BMI ***	24.35 (2.94)	23.97 (3.16)	Weight divided by height squared
GLU ***	106.28 (25.48)	99.74 (20.78)	Glucose
HBA1C ***	5.97 (0.92)	5.85 (0.74)	Hemoglobin A1c
TC ***	189.4 (35.35)	196.21 (35.39)	Total cholesterol
HDL ***	46.78 (11.18)	52.76 (12.07)	High-density lipid cholesterol
TG ***	167.91 (134.62)	125.17 (78.93)	Triglyceride
AST ***	25.02 (12.59)	21.83 (9.18)	Aspartate aminotransferase
ALT ***	25.19 (16.71)	19.29 (13.09)	Alanine aminotransferase
HGB ***	15.1 (1.23)	13.2 (1.13)	Hemoglobin
HCT ***	44.59 (3.37)	39.79 (2.98)	Hematocrit
BUN ***	15.85 (4.71)	14.57 (4.28)	Blood urea nitrogen
CRT ***	0.98 (0.37)	0.72 (0.23)	Creatinine
WBC ***	6.58 (1.82)	5.84 (1.63)	White blood cell
RBC ***	4.8 (0.41)	4.35 (0.33)	Red blood cell
FVC ***	4.17 (0.7)	2.96 (0.5)	Forced vital capacity
FVCP ***	91.83 (11.82)	94.47 (11.58)	Predicted forced vital capacity predicted
FEV1 ***	3.12 (0.67)	2.35 (0.45)	Forced expiratory volume in 1 s
FEV1P ***	90.24 (13.86)	94.51 (12.73)	Predicted forced expiratory volume in 1 s predicted
FEV1FVC ***	0.74 (0.08)	0.79 (0.06)	Ratio of forced expiratory volume in 1 s to forced vital capacity
FEV6 ***	3.99 (0.72)	2.89 (0.5)	Forced expiratory volume in 6 s
FEF25–75 ***	2.69 (1.2)	2.42 (0.83)	Forced expiratory flow 25–75%
PEF ***	8.49 (1.91)	6.08 (1.21)	Peak expiratory flow
SBP ***	122.26 (15.66)	119.52 (17.23)	Systolic blood pressure
DBP ***	78.16 (10.29)	74.69 (9.7)	Diastolic blood pressure

The values are expressed as means and standard deviations; Experimental results are presented as independent *t*-tests to verify statistical differences between two groups of men and women; and the statistical significance criteria are as follows: **: *p* < 0.01, ***: *p* < 0.0001.

**Table 2:** Results of statistical analyses of differences between normotension and prehypertension in men and women.

	Men		Women	
Features	*p*	OR (95% CI)	*p*	OR (95% CI)
HT	0.6494	0.978 (0.890–1.074)	0.1333	0.939 (0.866–1.019)
WT	<0.0001	1.364 (1.241–1.500)	<0.0001	1.348 (1.248–1.455)
WC	<0.0001	1.318 (1.206–1.441)	<0.0001	1.385 (1.281–1.498)
WHTR	<0.0001	1.335 (1.219–1.462)	<0.0001	1.424 (1.313–1.545)
BMI	<0.0001	1.429 (1.303–1.567)	<0.0001	1.427 (1.321–1.542)
GLU	0.0042	1.142 (1.042–1.251)	<0.0001	1.289 (1.180–1.410)
HBA1C	0.1937	1.059 (0.970–1.157)	<0.0001	1.225 (1.126–1.333)
TC	0.0102	1.122 (1.027–1.225)	<0.0001	1.229 (1.139–1.326)
HDL	0.0035	1.138 (1.043–1.242)	0.0381	0.924 (0.857–0.995)
TG	0.0001	1.203 (1.093–1.323)	<0.0001	1.259 (1.162–1.365)
AST	0.0006	1.193 (1.078–1.320)	0.0990	1.070 (0.987–1.160)
ALT	0.0004	1.187 (1.078–1.308)	<0.0001	1.191 (1.092–1.300)
HGB	<0.0001	1.322 (1.204–1.452)	<0.0001	1.166 (1.080–1.259)
HCT	<0.0001	1.262 (1.151–1.383)	0.0001	1.159 (1.074–1.250)
BUN	0.1314	0.933 (0.854–1.020)	0.1414	0.943 (0.872–1.019)
CRT	0.6373	0.979 (0.898–1.068)	0.8151	0.991 (0.921–1.066)
WBC	0.0673	1.084 (0.994–1.183)	<0.0001	1.216 (1.126–1.313)
RBC	0.0009	1.168 (1.065–1.281)	<0.0001	1.285 (1.191–1.385)
FVC	0.5738	1.029 (0.931–1.137)	<0.0001	0.827 (0.759–0.902)
FVCP	0.8888	0.993 (0.909–1.085)	<0.0001	0.814 (0.755–0.877)
FEV1	0.6471	1.026 (0.916–1.150)	0.0004	0.846 (0.770–0.928)
FEV1P	0.3469	1.042 (0.955–1.138)	0.0016	0.886 (0.822–0.955)
FEV1FVC	0.8762	1.007 (0.912–1.113)	0.3055	1.042 (0.962–1.129)
FEV6	0.7524	1.017 (0.914–1.130)	<0.0001	0.817 (0.747–0.893)
FEF25–75	0.9208	0.994 (0.891–1.109)	0.6338	1.021 (0.937–1.112)
PEF	0.9393	1.003 (0.908–1.109)	0.1210	0.937 (0.864–1.017)

The results of binary logistic regression analyses adjusted by age (*p* value, OR and 95% CI) for each feature in men and women are shown. Abbreviations: OR, odds ratio; CI, confidential interval.

**Table 3:** Results of statistical analyses of differences between normotension and hypertension in men and women.

	Men		Women	
Features	*p*	OR (95% CI)	*p*	OR (95% CI)
HT	0.0139	0.896 (0.822–0.978)	0.0048	0.883 (0.810–0.963)
WT	<0.0001	1.745 (1.592–1.912)	<0.0001	1.768 (1.627–1.921)
WC	<0.0001	1.789 (1.637–1.955)	<0.0001	1.927 (1.764–2.105)
WHTR	<0.0001	1.902 (1.734–2.086)	<0.0001	2.071 (1.884–2.276)
BMI	<0.0001	1.993 (1.817–2.185)	<0.0001	2.033 (1.861–2.221)
GLU	<0.0001	1.363 (1.247–1.489)	<0.0001	1.675 (1.508–1.861)
HBA1C	<0.0001	1.248 (1.148–1.357)	<0.0001	1.539 (1.393–1.700)
TC	0.0085	0.897 (0.827–0.972)	0.6831	0.984 (0.912–1.061)
HDL	0.5808	1.022 (0.944–1.106)	0.0001	0.857 (0.792–0.926)
TG	<0.0001	1.434 (1.304–1.576)	<0.0001	1.423 (1.306–1.551)
AST	<0.0001	1.341 (1.212–1.485)	<0.0001	1.238 (1.134–1.350)
ALT	<0.0001	1.310 (1.198–1.432)	<0.0001	1.453 (1.317–1.602)
HGB	0.0001	1.175 (1.081–1.278)	<0.0001	1.251 (1.151–1.359)
HCT	0.0055	1.123 (1.034–1.220)	<0.0001	1.205 (1.112–1.306)
BUN	0.4573	1.030 (0.951–1.117)	0.0836	1.076 (0.990–1.169)
CRT	0.0110	1.235 (1.049–1.453)	0.0001	1.334 (1.149–1.549)
WBC	<0.0001	1.183 (1.092–1.282)	<0.0001	1.347 (1.243–1.460)
RBC	0.0006	1.158 (1.064–1.260)	<0.0001	1.390 (1.283–1.505)
FVC	<0.0001	0.817 (0.744–0.898)	<0.0001	0.694 (0.630–0.764)
FVCP	<0.0001	0.791 (0.728–0.859)	<0.0001	0.681 (0.629–0.739)
FEV1	0.0263	0.888 (0.799–0.986)	<0.0001	0.753 (0.679–0.835)
FEV1P	0.3479	0.962 (0.888–1.042)	<0.0001	0.827 (0.763–0.895)
FEV1FVC	0.0046	1.138 (1.040–1.245)	0.0002	1.170 (1.076–1.272)
FEV6	<0.0001	0.806 (0.729–0.890)	<0.0001	0.682 (0.617–0.754)
FEF25-75	0.2276	1.063 (0.962–1.176)	0.2248	1.057 (0.966–1.157)
PEF	0.1416	0.933 (0.852–1.023)	0.5117	0.970 (0.888–1.060)

The results of the binary logistic regression analysis adjusted by age (*p* value, OR and 95% CI) for each feature in men and women are shown. Abbreviations: OR, odds ratio; CI, confidential interval.

**Table 4:** Details of the prediction models of prehypertension and hypertension.

Sex	BPS	FSM	Selected Features
Men	PHTN	CFS	WT, WHTR, BMI, GLU, AST, ALT, HGB
		WFS	LR: AGE, BMI, GLU, TC, HDL, TG, AST, HGB and BUN; NB: AGE, BMI, GLU, HDL, TG, AST and WBC; DT: HT, BMI, GLU, TG, WBC and FEV1;
	HTN	CFS	AGE, WC, WHTR, BMI, GLU, HBA1C, AST, CRT, FVC and FEV1
		WFS	LR: AGE, WHTR, BMI, GLU, TC, HDL, TG, AST, BUN, CRT, WBC, FVC, FEV1FVC, FEV6 and PEF; NB: AGE, WHTR, BMI, GLU, TC, HDL, AST, HGB and PEF; DT: AGE, WHTR, BMI, AST and FEV6;
Women	PHTN	CFS	AGE, WT, WC, WHTR, BMI, GLU, HBA1C, TC, TG, HGB, RBC, FVC, FEV1, FEV6 and PEF
		WFS	LR: AGE, WHTR, BMI, GLU, TC, TG, WBC, RBC and FVCP; NB: AGE, WT, WHTR, TC, TG, HGB, RBC, FVCP and PEF; DT: AGE, WHTR, BMI, GLU, HCT, RBC, FEV1 and FEV1P;
	HTN	CFS	AGE, WC, WHTR, BMI, GLU, HBA1C, TG, ALT, CRT, RBC, FEV1, FEV6 and PEF
		WFS	LR: AGE, WC, BMI, GLU, TC, HDL, TG, ALT, CRT, WBC, RBC, FVC, FEV6 and FEF25–75; NB: AGE, BMI, TC, HGB, RBC and FVCP; DT: AGE, BMI, GLU and HGB;

Abbreviations: FSM, feature selection method; BP, blood pressure status; PHTN, prehypertension; HTN, hypertension; CFS, correlation-based feature selection; WFS, wrapper-based feature selection; LR, logistic regression; NB, naïve Bayes; DT, decision tree; AUC, area under the receiver operating characteristic curve.
